# Notes and News

**Published:** 1898-06-04

**Authors:** 


### Notes and News.

(Collected and Communicated.)

The honorary secretaries of the Prince of Wales's Hospital Fund have received from "A Lady," who requests her name to be withheld, the munificent donation of £1,000.

The Lord Mayor is to preside at a meeting at the Mansion House on Thursday, June 16th, at three p.m., in aid of the Building Fund of the Central London Ophthalmic Hospital.

A second edition of Dr. Cuff's "Lectures on Medicine to Nurses" will shortly be published by Messrs. J. and A. Churchill, who also have in hand a new work on Bacteriology, by Dr. Hewlett.

A charming convalescent home at Khandalla is available for patients from the European hospitals in Bombay, and a nice bedroom is set apart for nurses going up for a change from hospital work in the cities.

Dr. T. E. Hayward, M.O.H. for the Haydock Urban District, has drawn up as a supplement to his annual report a life-table for Haydock, based on the mortality of the ten years 1881-90, and other tables showing the vital statistics of the district.

The Essex Cycling Union of Woodford and district are organising a procession of cyclists for the purpose of attracting a large number of people to the Woodford Meet and Fancy Dress Carnival, which will be held on June 4th on behalf of the local hospitals and charities.

The medical equipment of the surgeons going to the front with the United States Army seems to be pretty well up to date, if we may judge from the fact that each surgeon's chest contains three pairs of rubber operating gloves, sterilised, and wrapped in rubber tissue ready for use. Whether the rubber or the Lisle-thread gloves are the best for the purpose is, perhaps, a question.

The difficulty which occurred in coming to a satisfactory diagnosis in regard to the first cases of plague in Calcutta is making people ask for the permanent establishment of a complete bacteriological laboratory there, and the appointment of an officer who could devote himself entirely to the work.

The opening of the children's wing of the Lowestoft Hospital was marked by a picturesque procession of flower-laden children dressed in white, who sang "We are but little children weak." The wing is connected to the main building by a long covered way, which forms a promenade for convalescents. The dado round the wards consists of a series of nursery rhymes illustrated in hand-painted tiles. The wing is the Jubilee gift of Alderman and Mrs. Youngman.

Last week, at Scarborough Police-court, a man was fined £1 for having sold beer by retail without an excise licence. The evidence showed that an official of the Inland Revenue purchased from the defendant four bottles, containing respectively ginger beer, hop ale, hop bitters, and hop stout. Analysis showed that the ginger beer contained a little less than 2 per cent. of proof spirit, the hop ale 2·3 per cent., the hop bitter 4·9 per cent., and the hop stout 5 per cent.

The following house appointments have been made at St. Thomas's Hospital: House physicians—R. W. C. Pierce, H. H. Scott, H. F. Shea, and R. H. Bell; house surgeons—J. F. McClean, H. J. Marriage, J. S. Hall, and H. H. Sanguinetti; assistant house surgeons—E. H. Cobb, A. C. Robinson, F. L. A. Greaves, and A. H. Greg; obstetric house physicians—H. T. M. Alford (senior) and L. Gilbert (junior); clinical assistants in the Throat Department—A. J. Grant and F. R. Martin; clinical assistants in the Skin Department—W. J. E. Davies and P. W. G. Sargent; clinical assistants in the Ear Department—F. H. Allfrey and H. P. Kennard: clinical assistants in the Electrical Department—S. H. Belfrage and W. Thornely.

At the recent annual meeting of the Association of American Physicians a paper was read by Drs. Vaughan and McClymonds on the Bacteriology of Cheese. The productions of 86 manufacturers were examined, about 50 of them being ordinary American cheeses. In every one of them they found an organism which was pathogenic to white mice, guinea-pigs, rabbits, and white rats. These organisms were of the colon group. In no sample, however, of old ripe cheese did they find any pathogenic organism. This ought to be satisfactory to the eaters of ripe cheese. It is also interesting to note that in cheeses they discovered a group of germs which were rapidly peptonising, so that, perhaps, there may be some truth in what has so often been said—namely, that the cheese digests the dinner.

The serious rioting which has occurred again and again in Calcutta, and the many murderous assaults which have been committed on ambulances and upon persons suspected of being engaged in inoculating against the plague, make one question what would have happened if the disease had really become epidemic, and it is hard to avoid the feeling that the time has come when an end should in some way be put to the present condition of universal tolerance, which leads the Government to lend its sanction to every kind of abomination for which any excuse can be trumped up on the plea of native custom or native religion. If the natives will stick to their ridiculous customs they should remember that to be decimated by cholera, plague, and famine every few years was one of them.

